# Cross Talks between CNS and CVS Diseases: An Alliance to Annihilate

**DOI:** 10.2174/011573403X278550240221112636

**Published:** 2024-03-04

**Authors:** Shivani Chib, Sushma Devi, Rishabh Chalotra, Neeraj Mittal, Thakur Gurjeet Singh, Puneet Kumar, Randhir Singh

**Affiliations:** 1 Department of Pharmacology, Central University of Punjab, Bathinda, Punjab, India;; 2 Department of Pharmacology, Chitkara College of Pharmacy, Chitkara University, Punjab, India;; 3 School of Pharmacy, Graphic Era Hill University, Dehradun, India

**Keywords:** Cardiovascular disorders, neurological disorders, Alzheimer’s disease, Parkinson’s disease, Huntington’s disease, oxidative stress

## Abstract

Cardiovascular and neurological diseases cause substantial morbidity and mortality globally. Moreover, cardiovascular diseases are the leading cause of death globally. About 17.9 million people are affected by cardiovascular diseases and 6.8 million people die every year due to neurological diseases. The common neurologic manifestations of cardiovascular illness include stroke syndrome which is responsible for unconsciousness and several other morbidities significantly diminished the quality of life of patients. Therefore, it is prudent need to explore the mechanistic and molecular connection between cardiovascular disorders and neurological disorders. The present review emphasizes the association between cardiovascular and neurological diseases specifically Parkinson’s disease, Alzheimer’s disease, and Huntington’s disease.

## 1 INTRODUCTION

Particularly in industrialized nations, neurological diseases (NDs) are among the prime factors responsible for mortality. Numerous factors like genetic, environmental, and ageing-related variables are the primary fundamental factors responsible for NDs and neurodegeneration [1]. Neural dysfunctions are malfunction in certain regions of the brain throughout the brain and neurotraumatic illnesses, neurodegenerative diseases, and neuropsychiatric disorders are three primary categories of NDs including Alzheimer’s disease (AD), Parkinson’s disease (PD), and Huntington’s diseases (HD), depression/anxiety, and/or cognitive impairment. In 2010, more than 35 million individuals worldwide were affected with Alzheimer's disease, one of the main neurodegenerative illnesses. After AD, PD is the second most frequent neurological disorder among the elderly [2]. Whereas, cardiovascular diseases (CVDs) are widespread disorders and among the top factors responsible for mortality globally [3]. Moreover, the incidences of CNS and CVS are growing rapidly which poses an additional load on the healthcare system as well as society [4].

According to the pathological viewpoint, the prospective majority of neurological and cardiovascular illnesses originate with neuroinflammation, activation of oxidative stress, and dysfunction of the mitochondrial and proteasome systems. These factors include the fact that both the brain and the heart are electrically active organs, have high energy needs, and have a limited potential to rejuvenate following cell death [5]. From a pathophysiological standpoint, co-morbidities including systemic inflammation, hyperlipidemia, hypertension, and ageing as well as other stresses have an influence on both cardiac and mental health issues. Genetic or epigenetic factors may affect the heart and brain in a similar pattern [6]. The links between the heart and the brain are evident and diverse. CVDs provide a necessary context for brain disorders such as stroke, dementia, cerebral small vessel disease, and cognitive impairment. Atrial fibrillation, for example, has been linked with an increased risk of dementia and silent cerebral damage in people who have never had a stroke. Due to decreased cerebral blood flow induced by a failing heart, heart failure has been linked to cognitive impairment and dementia. Mental problems and negative psychological factors, on the other hand, may contribute to the initiation and progression of CVDs. Individuals suffering from disorders such as schizophrenia, bipolar disorder, epilepsy, or depression are at a higher risk of developing cardiovascular disease. As a result, our overall understanding of the anatomical and functional relationships between the heart and brain is still limited. So, the current study emphasizes the complex interactions between CVDs and NDs. Common pathways and risk factors of CVDs and NDs are illustrated in Fig. (**1**).

## 2 RELATION BETWEEN NEURODEGENERATIVE AND CARDIOVASCULAR DISORDERS

### 2.1 Alzheimer’s Disease (AD) and Cardiovascular Disorders (CVD)

AD is a neurodegenerative condition that worsens over time and destroys brain cells. The most typical cause of dementia is AD and memory loss and gradual neurocognitive impairment are the hallmarks of AD [7]. Accumulation of amyloid (Aβ) plaques and intracellular neurofibrillary tangles in the cortical and limbic regions of the human brain is responsible for the pathogenesis of AD. Furthermore, as the condition progresses, the Aβ-peptide level is elevated in CNS and starts the invasion of microglia. Microglia become activated adjacent to the accumulation of Aβ and surface receptors encourage their removal. Microglia also activate the innate immune system to react to aggregation [8]. Pathological alterations in amyloid precursor protein (APP) occur in AD with altered cleavage. APP is a protein that is essential for the plasma membrane in the central nervous system, and upon cleavage by β- and γ-secretase, APP is converted into insoluble Aβ fibrils [9]. This Aβ further oligomerizes and interferes with synaptic signaling when it diffuses into synaptic clefts and finally polymerizes into insoluble amyloid fibrils to form plaques. The microtubule-associated protein is hyper-phosphorylated due to kinases that are activated by this polymerization. The *Tau* (τ) proteins promote polymerization to form neurofibrillary tangles (NFTs) which are insoluble [10, 11]. Plaques and tangles forming together cause microglia to congregate around them, which leads to the precipitation of local inflammatory response, activation of microglia, and neurotoxicity. These neurons may develop large masses of NFTs and accumulate τ filaments. AD comprises distortion in the cellular system and neuronal death due to protein misfolding. Moreover, proteins lose their functions due to improper protein folding and abnormal protein aggregation also affects routine functioning leading to deleterious biological responses [12].

Ageing-related cellular and molecular changes such as inflammation have a significant role in the development of CVDs and AD [13]. Because there are common biological mechanisms, epidemiological studies have shown a relationship between the prevalence of CVDs and the advancement of dementia. Clinical and experimental reports suggest that Aβ peptides may function as a link between ageing, CVDs, and AD [14]. Organ malfunction and tissue inflammation are crucial parts of the AD and CVD theory and are caused by vascular and cardiac deposition of Aβ. Furthermore, with ageing, conditions like CVD and dementia get more [15].

Presently, it has become more evident that a person's predisposition to CVD may lead to an increased risk of cognitive decline in the future. After, vascular disorders are managed properly, the prevalence of dementia in both healthy and cognitively declined individuals may be attenuated [16]. All forms of dementia become worse with intracerebral atherosclerotic disease and result in decreased cognitive performance even in those who do not have dementia [17]. According to previous studies, age-related inflammation has several common cellular and molecular pathways that affect both dementia and atherosclerosis [18]. It has been found that aging-related disorders, such as atherosclerotic cardiovascular disease and neurodegenerative diseases, may be triggered by a common biological pathway that is currently under investigation [19].

**Fig. (2):**
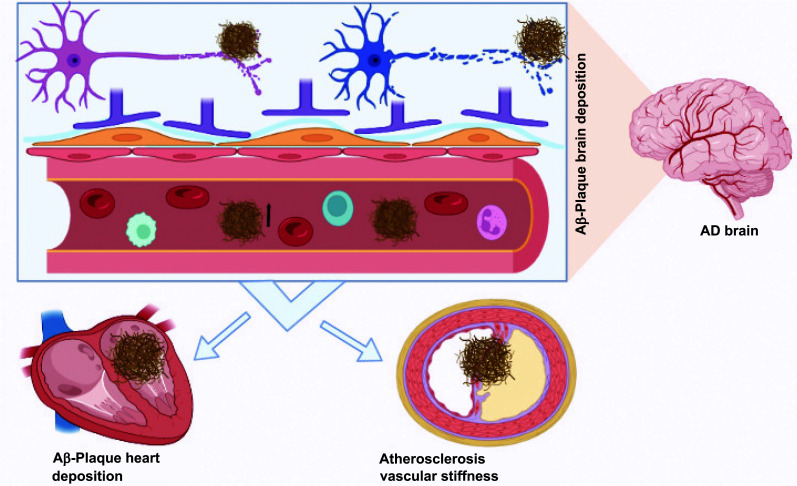
Cardiovascular and Neurotoxic Effects of Aβ in Brain parenchymal and cardiac. Brain Αβ deposits initiate many biological steps that associated in neurons dysfunctioning and manifested as diminish cognitive ability and potentiate Alzheimer. The depositions in cardiac are accompanying with cardiomyocyte dysfunction. Increase vascular stiffening, vascular inflammation and atherosclerosis. Cardiac dysfunctioning leads to cerebral hypoperfusion, Alzheimer, or dementia.

Aβ production and elimination are in equilibrium in the central nervous system, under normal circumstances. Ageing, inflammation, kidney disease, ischemia, polymorphisms, and medications increase the circulation and deposition of Αβ plaques either by increasing the generation and processing of APP or by reducing the clearance and deterioration of Aβ [14]. Deregulation of this balance may result in the buildup of Aβ_1_ in the tissues of the heart, vascular walls, and blood, which is linked to CVDs (Fig. **2**) [20].

Endothelial cells, macrophages, platelets, and vascular smooth muscle cells are affected by the activity of Aβ, which is a strong proinflammatory, proapoptotic, and proatherogenic chemical. The investigational research demonstrated that the dynamic involvement of Aβ peptides in the pathological processes results in plaque rupture, thrombosis, and coronary syndrome [21]. Αβ encourages platelet adhesion and activation whereas to promote the S2plaque rupturing, matrix metalloproteinase is secreted from human monocytes [22]. Small arterioles are less able to dilate when the amyloidogenic pathway is activated. All these alterations due to Αβ are confirmed in the histology of the heart with ischemic heart failure patients with AD [23]. The Apolipoprotein E (APOE) gene has been a subject of significant interest in Alzheimer's research due to its association with the risk and progression of the disease. APOE exists in three main alleles: APOE ε2, APOE ε3, and APOE ε4. Among these, APOE ε4 is considered a major genetic risk factor for late-onset AD. Individuals with one copy of the APOE ε4 allele have an increased risk, and those with two copies have an even higher risk [24]. APOE is involved in lipid metabolism and plays a crucial role in the transport of cholesterol and other lipids in the brain. Its deletion could disrupt these processes, potentially impacting neuronal function and the clearance of amyloid beta, a protein associated with AD pathology. APOE deficiency has been shown to affect synaptic function, increase vulnerability to neuronal injury, and alter inflammatory responses in the brain [25]. However, the precise molecular mechanisms and the extent of this impact require further investigation.

Dementia-prone APP23 mice mated with atherosclerosis-prone apolipoprotein E-deficient (ApoE-/) mice have larger and more inflammatory aortic atherosclerotic lesions than pure ApoE-/- mice [26]. It has been found that APP-deficient mice have considerably smaller plaques in the thoracic and abdominal aortas than ApoE-deficient mice, even if their cholesterol levels are the same [27]. So, atherosclerosis at various stages, and the development of clinically evident atherosclerosis CVDs may directly or indirectly be impacted by Aβ [28].

### 2.2 Parkinson's Disease (PD) and Cardiovascular Disorders (CVD)

Dopaminergic neurons in the substantia nigra are the primary targets of PD. Due to the loss of dopaminergic neurons, dopamine level is decreased in the basal ganglia [29]. Numerous risk factors associated with CVDs have been found to have relationships between environmental or genetic factors and PD. The pathways of glucose metabolism, cellular stress, lipid metabolism, metal toxicity, inflammation and others indicate common mechanisms or pathways of pathogenesis [30]. Instead, it appears that low-density lipoprotein cholesterol and cardiovascular disease have different relationships with PD [31]. The treatment of cardiovascular risk factors may therefore have no effect on the onset or progression of PD. Recent research has focused on the potential link between cardiac illness and PD, but only in terms of cardiovascular co-morbidity and cardiac autonomic dysfunction in PD patients. Cardiomyopathy, arrhythmias, coronary heart disease, and sudden cardiac death can occur in PD patients. PD patients reportedly have a higher-than-average rate of heart failure [32].

The pathophysiology of PD is characterized by elevated levels of circulating cytokines such as IL-1β, IL-2, IL-10, IL-6, IL-4, TNF-α, C-reactive protein, and interferon-gamma (INF-γ). These indicators are associated with oxidative stress and may potentially enable early PD diagnosis [33]. The majority of these circulating inflammatory mediators bind to platelet receptors, leading to platelet hyperactivity and aggregation that results in hypercoagulability or abnormal clotting potential in arteries. RBCs also experience programmed cell death known as eryptosis as a result of ligand binding and oxidative stress [34].

It is very evident that both chronic CD and PD share dysregulated pathways, inflammation, and metabolism, even if the connection between CVD and PD has not been thoroughly proven in earlier research [30, 31]. The risk of both CVDs and PD is hypothesized to be increased by cardiovascular risk factors including hyperglycemia, resistance to hormones like insulin, mild inflammation, excessive formation of reactive oxygen free radicals, and end products obtained after glycation end products. Nigrostriatal dopaminergic neurons are particularly vulnerable to hyperglycemia because they have many mitochondria, low glutathione levels, and high levels of iron ions which promote the production of highly reactive free hydroxyl radicals [35]. Moreover, oxidized LDL have a significant role in developing atherosclerotic plaques. Arginase, which contests nitric oxide present in the endothelium for arginine and reduces the bioavailability of nitric oxide, is expressed more frequently due to oxidized LDLs. This increases the development of atherosclerosis. It is unclear which pathway is responsible for the elevated oxidized LDL levels that play an important role in the development and/or progression of idiopathic PD [36]. Sphingolipid ceramide is related to PD, mild inflammation, cardiovascular disease, and insulin resistance. However, the accumulation of this sphingolipid hinders insulin action and works as a modulator of mitochondrial and ER stress [37].

CVDs and PD both originate and advance due to inflammation and are linked to persistently high C-reactive protein levels. In CVDs, inflammation plays a significant role in unstable plaque rupture as well as early leukocyte recruitment to the artery wall. Inflammatory cytokines, apoptosis, endothelial nitric oxide synthesis inhibition, stimulation of vascular cells, and thrombosis are possible outcomes of C-reactive protein activation. The meistic target of rapamycin signaling might be activated by C-reactive protein and TGFα/Smad3 pathways, which could lead to an increment of renal fibrosis and lead to diabetes and increase the risk of PD [38, 39].

### 2.3 Huntington's Disease (HD) and Cardiovascular Disorders (CVD)

HD is an autosomal dominant neurodegenerative condition in which there is a significant loss of neurons in the brain and striatum. HD is very distressing and progressive by nature. The prevalence of HD is 0.38 per 100,000 persons in a year [40]. Prior studies on HD have focused on how brain abnormalities lead to increased physical dysfunction, cognitive deterioration, and mental issues. Although HD is a well-known condition and mutant huntingtin protein (mHTT) is a key player in its pathogenesis, the mechanisms of neuronal death are yet unknown. Huntingtin protein (HTT) and mHTT are known to interact with several proteins and serve important roles in various cell types [41]. Surprisingly, individuals with HD frequently experience a significant occurrence of cardiac events, with heart failure ranking as the second most common cause of death among HD patients, contributing to 20–30% of HD-related deaths. Epidemiological investigations have indicated an association between HD and cardiac dysfunction, encompassing conditions such as cardiac amyloidosis and is marked by the misfolding of proteins and the buildup of aggregates within the heart [42]. A study on Drosophila substantiates that, the introduction of pre-amyloid oligomers (expanded PolyQ without HTT) specifically in cardiac cells has been demonstrated to result in cardiac abnormalities [43].

HD is a neurodegenerative disorder primarily known for its effects on the central nervous system, particularly in the striatum of the brain. However, emerging evidence suggests that the HTT gene, mutated in individuals with HD, is also expressed in peripheral tissues, including the heart. This raises intriguing questions about the potential impact of HTT dysfunction on cardiac physiology and the development of heart abnormalities in HD patients [44]. The HTT gene encodes the huntingtin protein, which is ubiquitously expressed throughout the body. Recent studies have confirmed the expression of HTT in the heart, implying a potential direct role in cardiac function [43]. The precise functions of huntingtin in the heart are not fully elucidated, but it is postulated to be involved in various cellular processes, including signal transduction, vesicular transport, and mitochondrial function [44]. Numerous studies have proposed that the cardiac dysfunction observed in HD patients may be attributed to dysfunction in the autonomic nervous system [45]. These findings indicate that the heightened risk of cardiac disease in HD patients may be linked to the accumulation of cardiac amyloids, coupled with other factors such as mitochondrial abnormalities and oxidative stress. Additional investigations involving mouse models of HD have also reported cardiac phenotypes, although aggregates in cardiomyocytes were not universally observed. Moreover, results suggest that the expression of mutant HTT protein with expanded PolyQ in mice neurons induces cardiac defects by affecting various pathways, including oxidative stress, mitochondrial dysfunction, and cell death [45].

Moreover, a neurotransmitter found in the brain and heart, called glutamate, plays a crucial function in cellular physiology. Glutamate metabolism may have a role in the etiology of cardiometabolic illnesses. Preclinically, the release of glutamate from the corticostriatal circuit was studied during the progression of the disease [46-53]. Glutamate and GABA abnormalities in HD can be attributed to abnormal dopamine transmission. Increased glutamate is released early in the progression of the disease. Reduced dopamine tone and/or loss of corticostriatal terminals are associated with reduced glutamate release by medium-sized spiny neurons in late-stage HD [54]. Recent molecular studies have revealed how glutamate is transferred across the cardiac mitochondrial membrane and is considered one of the probable mechanisms of glutamate transporters in mitochondria. It was found that the excitatory amino acid transporter (glutamate transporter), which is similar to the brain's glutamate transporter, is expressed in the heart [55]. However, glutamatergic neurotransmission disruption may be a key player in the pathogenesis of HD, particularly in terms of the unpleasant symptoms and cognitive deficits connected with the condition. As a result of this common mechanism, HD patients become more susceptible to cardiac disorders [56].

In HD, dopamine also interacts with glutamate and loss of dopamine input causes akinesia, whereas presynaptic stimulation of the nigrostriatal dopamine pathway causes chorea. A lack of GABA, which typically controls dopamine release by activating GABA receptors on nigrostriatal somata and terminals, may lead to overactivity in the nigrostriatal system in addition. Moreover, dopamine levels and the action of tyrosine hydroxylase are also increased up to the levels of the striatum of HD brains [57]. Diabetes is seven times as common in HD patients than in matched controls. Patients with HD have greater levels of insulin resistance, which results in decreased levels of insulin sensitivity [58, 59]. Another investigation highlighted the correlation between mutant HTT and cell death pathways, particularly evident in cardiac tissue. The study observed the activation of the Fas-dependent apoptotic pathway in the hearts of R6/2 HD mice. The researchers identified increased activity of key components associated with Fas-dependent apoptosis, including TNF-α, Fas ligand, Fas death receptors, FADD, activated caspase-8, and activated caspase-3, in the HD model compared to age-matched control mice. In addition to Fas-dependent autophagy pathway activation, the study revealed heightened activity of crucial components related to mitochondria-dependent apoptosis, such as Bax, Bax-to-Bcl-2 ratio, cytosolic cytochrome c, activated caspase-9, and activated caspase-3, in HD mice compared to wild-type mice. The collective findings suggested that the activation of both Fas-dependent and mitochondria-dependent apoptotic pathways could contribute to abnormal myocardial architecture, increased cardiac interstitial spaces, and a higher number of cardiac TUNEL-positive cells in HD mice [45]. The association between the risk factors of CD and HD has been illustrated in Fig. (**3**).

## 3 COMMON RISK FACTORS BETWEEN CVS AND CNS DISEASES

Woefully, several neurological disorders have credence as having a bidirectional association with cardiovascular morbidity. Numerous factors involved in the pathogenesis of CNS disorders and associated CVS outcomes have been mentioned in the literature. In further snippets, the various risk factors involved in the pathogenesis of both CVS and CNS diseases are described.

### 3.1 Insulin Resistance

Interestingly, insulin resistance is not merely associated with a deficiency in glucose uptake, but a multifaceted syndrome that ameliorates the risk of CVS and CNS disorders. Insulin plays a critical role in maintaining homeostasis in the periphery and CNS. The disruption in insulin signalling may increase the risk factor of CVS disorders (arteriosclerosis, cardiomyopathy, dyslipidemia, coronary artery disease, and hypertension) and CNS disorders (AD, depression, PD, Schizophrenia, psychosis and epilepsy) [60-65]. Insulin plays a significant role in the brain and all cells of the CNS have insulin receptors (IR) with high density in CA1, and CA3 neurons of the hippocampus, dentate gyrus, hypothalamus, cerebral cortex and striatum [66]. Alterations in insulin signalling affect neuronal plasticity and brain ageing. Contrastingly, IR is also expressed in cardiomyocytes and vasculature. Insulin signalling modulates cardiomyocyte functions such as cell survival and growth and reduces autophagy and apoptosis probably *via* PI3K/AKT pathway [67]. Upon activation, the IR presence of vascular endothelial cells induces eNOS phosphorylation, resulting in NO production and subsequent vasodilation [68]. The study observed that impaired insulin signalling leads to decreased heart size during development [69]. Moreover, accelerated left ventricle remoulding and pressure overload were found in mice lacking cardiomyocyte insulin receptors [70]. Constitutive deletion of insulin receptors on cardiomyocytes impaired mitochondrial respiration capacity and ATP production [71].

Nevertheless, insulin resistance certainly adds to the risk of hypertension. Researchers observe a significant relationship between high blood pressure and high insulin levels, probably due to abnormalities in vasodilation and blood flow [72]. Increased insulin levels elevate the reabsorption of sodium and water by tubular cells of the kidneys, thus increasing the risk of volume-dependent hypertension [60]. Studies found the overactive sympathetic system in insulin-resistant patients, is another causative factor in insulin-mediated hypertension [72]. In adipocytes, insulin resistance increases the release of free fatty acids (FFA) into circulation, which in turn increases the VLDL and LDL levels and alleviates the availability of HDL [60]. All these consequences critically contribute to the pathophysiology of CVS and CNS disorders including atherosclerosis and AD.

In the case of CNS disorders, insulin resistance is predisposed to the pathophysiology of various neurological disorders, where depression-induced chronic stress increases the release of corticotropin-releasing factor (CRF) and activates the hypothalamus-pituitary axis (HPA) which elevates glucocorticoid levels. These glucocorticoids increase the production of proinflammatory cytokines such as interleukins (IL), Tumor necrosis factor-alpha (TNF-alpha) and decrease BDNF activity [73]. Furthermore, an increased level of glucocorticoids elevates hepatic gluconeogenesis resulting in hyperglycemia and subsequent insulin resistance [74]. Glucocorticoids are reported to disrupt the activity of pancreatic beta-cells by alleviating the expression of GLUT2 and glucokinase. Moreover, it also reduces insulin sensitivity by decreasing the expression of insulin response substrate 1 (IRS1) [75]. Glucocorticoids are also thought to play an imperative role in AD pathology. Insulin resistance is another hallmark of AD [76]. Notably, glucocorticoids increase the production of Aβ probably by elevating the level of APP and beta-site amyloid precursor protein cleaving enzyme 1(BACE1). In addition, insulin resistance potentiates the function of GSK-3β, which increases the phosphorylation of Tau and the formation of neurofibrillary tangles, which contributes to AD pathology. Furthermore, the level of GSK-3β and PTEN was found to increase along with a significant decrease in IRβ, IRS1, GLUT1, GLUT4, PI3K p85α and pAkt1/2/3 level in the hippocampus and nuclear fraction of Genetic Absence Epilepsy Rats from Strasbourg [63]. Moreover, impaired insulin signalling is also reported in PD. Chen Tai Hong and colleagues found insulin resistance results in increased expression of α-Synuclein (SNCA) and ROS in dopaminergic neurons, which leads to their degradation. In addition, insulin resistance causes hyperactivity of PLK-2, pSNCA and k-resistance SNCA levels in SH-SY5Y cells of PD and PLK2 inhibitors reverse the insulin resistance-mediated abnormalities [64].

### 3.2 Cytokines and Chemokines

Cytokines (TNF-α, IL, IFN, and TGF) and chemokines (CCL, CX3CL, CXCL, CXC, and XCL) are secreted proteins that regulate both cellular and innate immune responses. Notwithstanding their protective role, myriad evidence suggests that these small proteins play a central role in the pathogenesis of various CVS and CNS disorders. Depending on the immune cells present, cytokines have both anti-inflammatory and pro-inflammatory potential [77]. In the context of CVD, atherosclerosis gains centrality because it precedes myocardial infarction, stroke, and heart failure. CXC chemokine ligands are released from endothelial cells through lysophosphatidic acid (a component of LDL) and recruit leukocytes, monocytes, and neutrophils to the site during atherogenesis. Along with chemokines, inflammatory cytokine also contributes to the formation of atherosclerotic plaque formation. Chemokine such as CCL5 has an important role in the development of atherosclerosis and is used as a biomarker in various studies to quantify the risk of CVD [78, 79]. In the line, inhibition of CX3CR1 shows potential for early atherosclerotic lesions and alleviates monocyte adhesion [80]. Nevertheless, the level of proinflammatory cytokines such as TNF-α, IL-1β, IL-6, and IFN-γ was also found to be elevated in several heart diseases including congestive heart failure, atherosclerosis, coronary artery disease and myocardial infarction [81, 82]. Canakinumab Anti-inflammatory Thrombosis Outcome Study revealed that the deactivating cytokine IL-1β reduced inflammation and cardiovascular death in myocardial infarction patients [83].

Besides, CNS also comes under the vulnerable grip of both cytokines and chemokines. Numerous studies confirmed the pronounced role of inflammation in neurological disorders and these small proteins play a lead role in inflammatory cascade in CNS. Factors responsible for neurological disorders such as Aβ, OS, NFT, α-synuclein, and others initially cause microglial activation, which further leads to the release of inflammatory cytokines and worse pathology of the disease [84]. In line, Aβ activates microglia in a mouse model of AD and 21 days of treatment with matrine, an anti-inflammatory drug, that reduces the concentration of TNF-α, IL-1β, and IL-6, thus protecting the cognitive deficit [85]. In animal models of PD, haloperidol caused the elevation of inflammatory cytokines which lead to motor deficit and dopaminergic neuronal degradation and 35 days of treatment with lauric acid reversed these consequences [86]. In addition, oral administration of andrographolide decreased the level of chemokines such as CCL2, CCL5, CXCL1, CXCL2, and CXCL9 in LPS-induced neuroinflammation, thus protecting the mouse brain [87]. Conclusively, both cytokines and chemokines are significant contributors to CVS as well as CNS disorders. However, short-term expression of both is needed to maintain homeostasis inside the body.

### 3.3 Brain-Derived Nerve Growth Factor (BDNF)

BDNF is a protein that regulates the growth, differentiation, and survival of neurons in the periphery as well as CNS. BDNF utilizes the saturable transportable system to cross the BBB. The major role of BDNF is to maintain synaptic plasticity and memory formation [88]. Cortical BDNF levels exhibit a strong correlation with serum BDNF concentration in the periphery. In contrast to neurons, peripheral endothelial cells also contain BDNF and its Trk-receptors [89]. BDNF has also been shown to play a predisposing role in CVS disease, especially through blood pressure and heart function regulation [90]. Most often, low levels of BDNF have been associated with a higher risk of hypertension and other CVS diseases [91]. This notion is supported by a study in which the plasma level of BDNF was found to be decreased in patients with acute coronary syndrome and DM [92]. Pressure load-induced cardiac hypertrophy disrupts the baroreflex *via* the heart-to-brain axis probably due to an increase in TRPV1 in the heart and BDNF in NTS [93]. In line, Andrea *et al.* found that ageing alleviates circulating and cardiac BDNF levels in rats and depletes the cardiac autonomic nerve fibres [94]. A low level of plasma BDNF is associated with prognosis in patients with Angina Pectoris [95]. Moreover, in a clinical study on patients with coronary heart disease, plasma BDNF level was found to be reduced significantly compared to the control group [96].

Notwithstanding, neurological disorders have also been linked to reduced or lack of expression of BDNF in the brain. BDNF has an affinity for Trk receptor, followed by modulation of MAPK, AKT/PI3K, and PLC- γ pathways. The former activates the transcription factor CREB, which in turn upregulates the genes responsible for neuronal growth and development [97]. Myriad evidence suggests that elevating the BDNF level potentiates learning and reduces Aβ-induced AD in a mouse model. Restoration of BDNF level improves behaviour, memory, synaptic degradation, and neuronal loss in P301L mice of AD [98]. In the context of PD, a low level of BDNF was found in both animals as well as patients’ nigrostriatal pathway and physical training improved the BDNF and Trk levels in PD animals [99]. Moreover, loss of neuronal plasticity was observed in depressed patients and further analysis revealed a decreased level of BDNF in the blood and brain samples of these patients [100]. In a mouse model of stress, loss of BDNF level was found in the hippocampus and prefrontal cortex, and similar results were also observed in social stress-induced depressed mice [101]. So, BDNF could be a potential therapeutic target for drugs against CVD and CNS diseases.

### 3.4 Other Influential Factors: Exploring Family History, Gender, Lifestyle and Disease Conditions

In context to family history, genetic mutations cause the development of CVD and ND and contribute to the risk of inheritance leading to high risk within the family or in close relatives, which further may result in increased risk of CVD and ND. If a family member experiences CVD before 55 years in men and 65 in women, there would be an increased risk of developing CVD in other family members, as families often share similar lifestyles, habits, diet, and exposure to environmental factors, that could lead to the risk of CVD. Similarly, certain neurological disorders like PD, AD, and certain types of epilepsy also have a genetic factor, that could pass through generations. In a study of 2302 participants with a follow-up of 8 years, there was a 75% increase in cases of CVD by paternal and about 60% increase in cases with a maternal history of premature CVD. In identical twins, the hazard ratio of death from coronary artery disease increased from 3.8 to 15 folds, *i.e.,* if an identical sibling died of CVD before the age of 75 [102]. In the case of ND, one cohort study of 567436 Swedish participants revealed that there is a high risk of persons with family background affected by these diseases [103].

Besides, age is a significant factor that influences the development of both CVD and ND. Age-related changes appear in people like the accumulation of plaques in arteries, decrease in their elasticity, and reduced efficiency in pumping blood, and these factors seem to have a direct effect on CVD [104]. Dementia and Alzheimer’s occur with age and are considered normal, other factors like amyotrophic lateral sclerosis, Parkinson’s disease, and ischemic stroke also have a high incidence in older patients, due to nerve and motor loss with age. Although age is considered a significant factor, that does not mean everyone would suffer from these diseases and an active lifestyle, and healthy diet may reduce the risk of diseases [105].

Moreover, the gender of an individual also influences the risk factors and conditions of CVD and ND, as each sex seems to be similar, but there are some changes like hormonal, neurological, and genetic ones. Men have a high risk of developing coronary artery disease at a young age, whereas women have a significant risk after menopause. Estrogen provides protective effects, vasodilation and reduced inflammation [106], women have unique risk factors related to pregnancy like gestational diabetes, and all these factors are related to CD. Whereas in ND, for instance, conditions like migraine, Alzheimer’s, and multiple sclerosis have different patterns in men as compared to women. Testosterone and estrogen have certain effects, like estrogen has shown a low risk of developing Parkinson’s in women, and testosterone also showed improved cognitive function [107, 108].

In addition, blood pressure plays a critical role in maintaining adequate blood flow to the brain. Proper blood pressure regulation ensures the brain receives an adequate blood supply to support its metabolic demands and optimal functioning. Chronic high blood pressure could damage blood vessels in the brain, leading to ischemia and increasing the risk of neurological impairment [109]. Similarly, blood pressure can cause damage to arterial walls, and blood vessels throughout the body, leading to atherosclerosis, arterial stiffness, and an increased risk of cardiovascular disease. Low blood pressure also leads to reduced blood flow in vital organs [110]. Furthermore, cigarettes contain nicotine, a highly addictive chemical substance, that stimulates the release of dopamine, prolonged smoking leads to dopamine addiction, which causes changes in neurotransmitter levels, smoking also develops neurological disorders, like stroke, Alzheimer’s, and Parkinson’s disease [111]. Furthermore, it has chemicals that damage the inner lining of blood vessels, promotes inflammation and vasoconstriction, leading to raised blood pressure, that could develop into atherosclerosis, stroke, and other heart diseases. Smoking also promotes blood clotting that could cause severe cardiovascular complications [112].

Interestingly, alcohol consumption depresses brain activity, leads to relaxation, and impairs judgement, motor coordination, and reaction time. It alters the neurotransmitter level in the brain like GABA, leading to a reduction in the excitatory effects of glutamate. It impairs cognitive functions like memory and attentiveness, and in some cases leads to brain damage [113, 114]. Furthermore, it showed effects on blood pressure, leading to long-term hypertension, irregular heart rate rhythm, elevated triglycerides, and even caused cardiomyopathy, along with other risk factors like ischemic and haemorrhagic strokes [115]. Also, lipids, especially phospholipids, are the major structural components of cell membranes. Nerves are surrounded by these lipids, namely, myelin. The myelin sheath facilitates the rapid conduction of nerve impulses along axons for efficient communication within the CNS. Lipids also play a major role in the formation of neural connections and neurogenesis [116]. Another lipid, cholesterol, is necessary for the production of steroidal hormones, bile acids, and vitamin D, however imbalance between HDL, and LDL could cause atherosclerosis, triglycerides are another lipid, and their elevated level in blood could cause cardiovascular diseases [117].

Moreover, people with diabetes or consistent diabetes are at high risk of developing dementia, and cognitive impairment, prolonged diabetes leads to diabetic neuropathy that cause symptoms like pain, numbness, and tingling in the extremities. Diabetes also affects the nerves that control bodily functions [118]. Diabetes is also a major risk factor for cardiovascular diseases, including coronary artery disease, heart failure, and stroke. Chronic diabetes could lead to endothelial dysfunction, inflammation, and oxidative stress that promotes the risk of cardiovascular events and could lead to cardiomyopathy. Hypertension is also most prevalent in diabetic patients which influences kidney problems [119]. Also, obesity is associated with an increased risk of cognitive impairment, excess weight has been linked to impairment in memory, execution of various functions, and processing of nervous stimuli. Obesity also affects the structure and functioning of the brain, like changes in brain volume, affecting the area for decision-making, impulse control, processing, control behaviours like overeating, and difficulty in controlling food ingestion [120]. It also has a role in depression, and anxiety, which further impact ND. Obesity also has a significant effect on cardiovascular diseases. Obesity is also a risk factor for type 2 diabetes and excessive adipose tissue also results in insulin resistance [121]. Moreover, regular physical activity has been associated with improving cognitive functions and reducing the risk of cognitive decline. Exercise regulates the release of growth hormones, promotes the formation of new neurons, and improves memory. Exercise also has been shown to reduce the risk of depression, alleviate symptoms of anxiety, improve overall mood, and stimulate the release of endorphins, neurotransmitters, that promote wellbeing and reduce stress. Exercise also increases neuroplasticity, a strengthening of neural pathways, that has a positive role in memory, learning, and overall brain function [122]. Furthermore, regular physical activity reduces cardiovascular risk by increasing heart strength, improving circulation, lowering blood pressure, and developing atherosclerosis. Regular physical exercise maintains the ratio of HDL *vs.* LDL, and regulation of blood sugar levels [123]. In the line, arterial fibrillation, in particular, increases the risk of stroke, and irregular arrhythmia can pool blood, leading to the formation of clots and ischemic stroke. Arterial fibrillation-related strokes can have significant neurological consequences that could impair cognitive functions. Factors like microemboli and reduced blood flow may contribute to cognitive impairment [124]. Furthermore, arrhythmia involving AF can disrupt normal electronic signaling of the heart, leading to irregular heart rhythms which affects the pumping of blood. Certain arrhythmias, like ventricular tachycardia or ventricular fibrillation, can cause hemodynamic instability that causes sudden loss of blood flow to vital organs, including the brain can cause cardiac arrest, and severe neurological consequences [125].

## 4 IMPACT OF MEDICAL TREATMENT AND LIFESTYLE CHANGES ON CARDIAC DISORDERS AND NEUROLOGICAL DISORDERS RISK FACTORS

Despite notable progress in medical technology and pharmacology, CVDs and NDs continue to be a significant contributor to healthcare expenditure and the primary cause of mortality worldwide. The reduction of the likelihood of recurrent events poses a challenge for healthcare providers in patients diagnosed with established CVD or ND. The implementation of “secondary prevention” can be accomplished through the favourable modification of major coronary risk factors, including but not limited to hypertension, hypercholesterolemia, diabetes, and obesity. Nevertheless, lifestyle modification can prove to be advantageous in this aspect, offering self-sufficient and supplementary advantages to the correlated declines in CD and ND morbidity and mortality. It is recommended that healthcare professionals advise patients to partake in organized physical activity and incorporate more physical activity into their daily routine, adhere to a diet that promotes heart and mental health, cease smoking, and steer clear of second-hand smoke, and deliberately manage psychosocial stressors that may heighten the risk of cardiovascular disease. The implementation of lifestyle interventions, either as a supplementary measure to medication therapy or as a standalone approach in cases where medication may not be well-tolerated, financially feasible, or efficacious, has been shown to considerably reduce mortality rates and the likelihood of recurring cardiac events. Table **1** summarizes the impact of medical treatment and lifestyle changes on CVDs and ND risk factors.

## 5 DISCUSSION

The interplay between cardiovascular and neurological disorders underscores the vital role of a healthy cardiovascular system in preserving neurological well-being. The cardiovascular system plays a pivotal role in maintaining cerebral perfusion and neuronal integrity [126]. Hypertension, atherosclerosis, cardiac arrhythmias, cardiac arrest, and chronic heart failure exert profound effects on cerebral blood flow, oxygenation, and nutrient supply, contributing to the development and progression of neurological complications [127]. A comprehensive understanding of these associations can guide the development of preventive strategies and targeted interventions aimed at reducing the burden of both CDs and NDs.

AD, PD, and HD symptoms can occur due to cardiac illness. According to research, 6-23% of all ischemic strokes are considered to be caused by cardiogenic embolism, which accounts for around 15% of all such cases. In recent clinical research, cardiogenic embolism was found to be responsible for one in six ischemic strokes [50]. Cardiac autonomic dysfunction is the most notable of the several cardiac abnormalities identified in PD and diagnosis of cardiac autonomic dysfunction in PD can be done using several methods. Coronary heart disease in people with PD may occur more frequently or less frequently depending on the country and the afflicted individuals' lifestyle [128, 129]. Research studies have demonstrated a link between cardiovascular risk factors such as oxidative stress, mitochondrial dysfunction, Aβ accumulation, impaired glucose metabolism, vascular dysfunction, and cerebral small vessel disease with the development of AD, PD and HD [130-132]. Furthermore, mutant huntingtin protein may directly affect cardiovascular function, leading to structural and functional abnormalities in the heart [133].

Inflammation plays a critical role in the initiation and progression of CVD and NDs. Pro-inflammatory cytokines, such as IL-6 and TNF-α, are elevated in both conditions and inflammation plays a significant role in the development of atherosclerosis, promoting endothelial dysfunction and plaque formation in cardiovascular disorders [134]. Similarly, neuroinflammation mediates neuronal damage and neurodegeneration in neurological disorders such as stroke, PD, and AD [135]. Furthermore, abnormalities in fatty acid metabolism lead to increased levels of circulating free fatty acids, triglycerides, and cholesterol, which in turn contributes to atherosclerosis and coronary artery disease [136]. Similarly, in neurological disorders, disturbances in fatty acid metabolism can impair mitochondrial function, compromise energy production, and contribute to neuronal dysfunction and neurodegeneration [137]. Insulin resistance, a hallmark of metabolic disorders like obesity and type 2 diabetes, is closely linked to both CVDs and NDs. Insulin resistance disrupts glucose homeostasis and impairs endothelial function, promoting a pro-atherogenic environment in CVS and impairing neuronal survival, synaptic plasticity, and cognitive function in the CNS [138]. Moreover, decreased levels of BDNF have been observed in individuals with CVDs and NDs such as AD and PD, contributing to neurodegeneration and cognitive decline [92, 93]. Age-related physiological changes, including vascular stiffness, endothelial dysfunction, and accumulation of amyloid -β in the heart and brain, contribute to the development and progression of both CVDs and NDs. In addition to this, unhealthy lifestyle choices, such as sedentary behaviour, poor diet, smoking, alcohol consumption, high-calorie, and high-fat diet contribute to obesity, hypertension, dyslipidaemia, and insulin resistance which ultimately leads to the development of CVDs and NDs. There are two modifiable risk factors, physical activity and moderate coffee consumption, that are inversely linked with CVDs and NDs; however, the pathways by which they are linked with NDs are not established, and research to date supports the preponderance of evidence indicating divergent pathways. These risk factors (or their underlying processes) can serve as primary or secondary prevention targets, regardless of diagnosis. People at risk of developing NDs can benefit from good glycemic management and the treatment of high blood pressure as supplementary health treatments, nevertheless a lack of clear epidemiological evidence in this regard for ND patients or those at risk of developing NDs. Overall, a number of factors exhibit association in the pathogenesis of CVDs and NDs. It is specifically advised that patients of NDs should have more thorough cardiological examinations to determine the likeliness of developing cardiological disorders.

## CONCLUSION

NDs and CVDs are major contributors to morbidity and mortality globally. Along with ageing, epigenetic and genetic factors play an important role in the pathogenesis of NDs and CVDs. A number of pathways have interplay in both NDs and CVDs and numerous biomarkers such as insulin, BDNF, cytokines, and chemokines exhibit common pathological facets in NDs and CVDs. These molecules potentiate the crosstalk between CNS and CVS *via* migrating from the brain to the periphery or vice versa during pathogenesis. For decades, these molecules have been credited as biomarkers for the diagnosis of various NDs and CVDs. The present work attempts to elucidate these biomarkers as prognostic markers for NDs if these are diagnostic markers for CVDs or *vice versa*. Therapeutic strategies to treat CDs and NDs may be devised to treat one disease considering the association with other diseases and will act as a double-edged sword.

## Figures and Tables

**Fig. (1):**
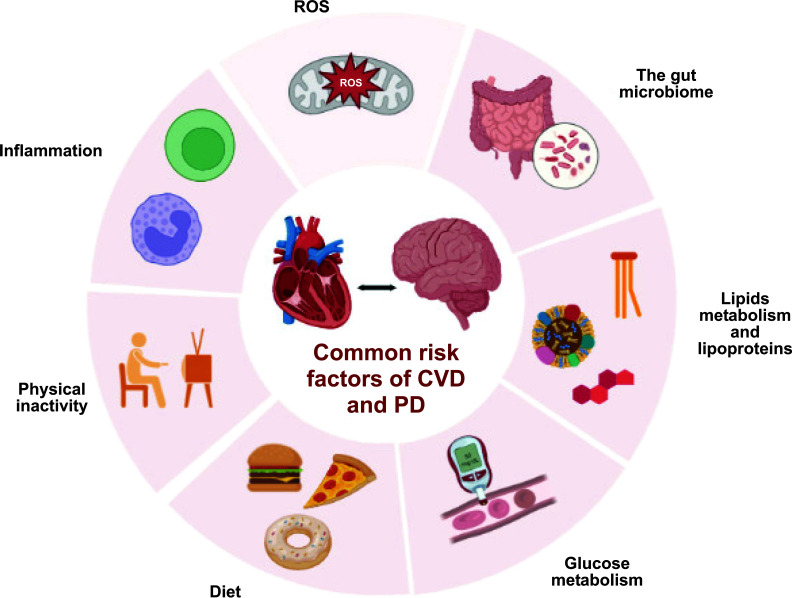
Common pathways and risk factors of CDs and PD. **Abbreviations:** ROS- Reactive oxygen species, ox-LDL-oxidized low-density lipoproteins.

**Fig. (3):**
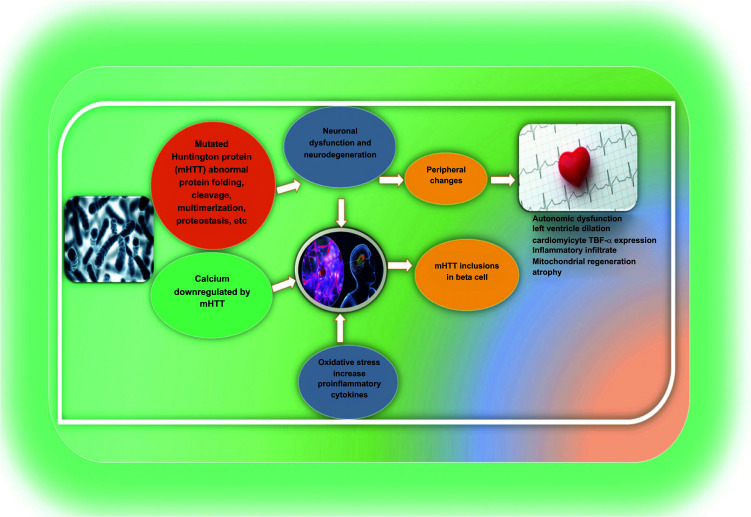
Brain-heart pathogenesis in Huntington’s disease.

**Table 1:** The impact of medical treatment and lifestyle changes on CDs and NDs risk factors.

**Risk Factor**	**Medical Treatment**	**Lifestyle Changes**	**Outcomes**
High Blood Pressure	Medications (*e.g.*, ACE inhibitors, beta-blockers) can help lower blood pressure levels.	Reducing sodium intake, adopting healthy diet (*e.g.*, DASH diet), regular physical activity, weight management, limiting alcohol consumption, managing stress.	Lower blood pressure levels, reduced risk of cardiovascular events (CAD, left ventricular hypertrophy, arrhythmia) brain stroke, vascular dementia and intracerebral haemorrhage.
High Cholesterol	Statin medications can lower LDL cholesterol levels and raise HDL cholesterol levels.	Following a heart-healthy diet (*e.g.*, low in saturated and trans fats), regular physical activity, weight management, limiting alcohol consumption, quitting smoking.	Lower LDL cholesterol levels, improved lipid profile, reduced risk of ischemic stroke, atherosclerosis and Alzheimer’s disease.
Diabetes	Medications (*e.g.*, metformin, insulin) can help regulate blood sugar levels.	Following a balanced diet (*e.g.*, low in refined sugars and carbohydrates), regular physical activity, weight management, blood sugar monitoring, stress management, quitting smoking.	Blood sugar control, reduced risk of CAD, arrythmias, neurological complications such as Alzheimer’s disease.
Obesity	Medications (*e.g.*, orlistat) may be prescribed in certain cases.	Healthy eating habits, portion control, regular physical activity, reducing sedentary behaviours, seeking support from healthcare professionals or weight management programs.	Weight loss, improved cardiovascular events, dementia, sleep-disorder, depression and anxiety
Smoking	Nicotine replacement therapy (*e.g.*, patches, gum) and medications (*e.g.*, bupropion) can aid in smoking cessation.	Quitting smoking, seeking support from healthcare professionals or smoking cessation programs, adopting healthier coping mechanisms, avoiding triggers.	Improved cardiovascular and neurological health, reduced risk of related diseases
Physical Inactivity	N/A	Incorporating regular physical activity into daily routine, reducing sedentary behaviours (*e.g.*, sitting for long periods), engaging in exercise or active hobbies.	Reduce risk of cardiovascular and neurological health.
Unhealthy Diet	N/A	Following a balanced diet rich in fruits, vegetables, whole grains, lean proteins, and healthy fats; reducing intake of processed foods, sugary beverages, and high-calorie snacks.	Improved cardiovascular and neurological health, reduced risk of related diseases
Excessive Alcohol Consumption	Medications may be prescribed in certain cases to address alcohol dependency.	Limiting alcohol intake, seeking support from healthcare professionals or alcohol addiction programs, practicing moderation or abstaining from alcohol.	Improved cardiovascular and neurological health, reduced risk of related diseases
Stress	Medications may be prescribed in some cases to manage stress-related conditions (*e.g.*, anxiety, depression).	Engaging in stress-reducing activities (*e.g.*, exercise, meditation, mindfulness), practicing relaxation techniques, seeking support from mental health professionals.	Improved mental well-being, reduced risk of cardiovascular and neurological diseases
Cognitive Stimulation (for neurological diseases)	Medications (*e.g.*, cholinesterase inhibitors for Alzheimer's disease) may be prescribed to manage symptoms.	Engaging in mentally stimulating activities (*e.g.*, puzzles, reading, learning new skills), social interactions, maintaining an active social and intellectual life	Enhanced cognitive function, improved brain health, reduced risk of neurological diseases.

## LIST OF ABBREVIATIONS

AD: Alzheimer’s Disease
APOE: Apolipoprotein E
APP: Amyloid Precursor Protein
CRF: Corticotropin-Releasing Factor
CVDs: Cardiovascular Diseases
FFA: Free Fatty Acids
HD: Huntington’s Diseases
HPA: Hypothalamus-Pituitary Axis
IR: Insulin Receptors
NFTs: Neurofibrillary Tangles
PD: Parkinson’s Disease
TNF-alpha: Tumor Necrosis Factor-alpha
τ: *Tau*
